# Heart Disease in Adult Syrian Refugees: Experience at Jordan University Hospital

**DOI:** 10.5334/aogh.2474

**Published:** 2019-03-14

**Authors:** Amjad Bani Hani, Mahmoud Abu Abeeleh, Moaath Al Smady, Mamoun Shaban, Murad Al Kharabsheh, Zahraa Al-Tamimi, Sandra Eifert

**Affiliations:** 1 General Surgery Department, School of Medicine, The University of Jordan, Amman, JO; 2 Dept. of Cardiac Surgery University Munich, DE

## Abstract

**Background:**

Since 2011, 1.26 million Syrians have immigrated to Jordan, increasing demands on Healthcare service. Information about cardiovascular disease (CVD) in Syrian refugees in general, and specifically in Jordan, is unknown.

**Objectives:**

The study aimed to describe CVD in Syrian refugee adults who were referred to Jordan University Hospital (JUH) in terms of diagnosis, presentation, outcome, sources of funding for treatment, and to follow these patients after their discharge.

**Methods:**

From January 2012 to October 2016, retrospective analysis was performed on the data of Syrian patients who were referred to JUH. This study describes the diagnoses, treatment, and outcome. It also discusses the funding sources; a follow-up was conducted until January 2017.

**Results:**

There were 969 patients referred to JUH with CVD; median age was 56 years, 686 (72.2%) of them were males and 283 (27.8%) were females. Of the patients, 584 had hypertension (60%), 308 (31%) had diabetes mellitus, 281 (29.0%) suffered from dyslipidemia, and 237 were smokers (24%). There were 69.6% who had coronary artery disease (CAD) and 20 patients (2%) had valvular heart disease. Treatment was offered to 489 patients (49.5%), but only 322 (65.8% of treatment offered and 33.2% of referrals) of them received the intended treatment. Mortality rate was 3% and loss of follow-up was 49.2%. Funding for procedures mostly came from the Jordanian Health Aid Organization, the United Nations, NGOs, and charities. Sixty-four (13.3% of referred) patients were denied any funding during the time frame of this study.

**Conclusions:**

CVD is a major issue for both Syrian refugee patients and the Jordanian healthcare system. CAD and classic cardiovascular risk factors (specifically arterial hypertension, diabetes, and dyslipidemia) are most common in this specific population. Inadequate primary healthcare, suboptimal living conditions, lack of funding, and loss of patient contact are among the major challenges facing this vulnerable population.

## Introduction

In the last decade, the Syrian refugee crisis has developed. Consecutively, millions of Syrians were forced to flee their country, seeking refuge primarily in neighboring nations like Turkey, Iraq, and Jordan. Host countries have since been exposed to increasing demands in all aspects of life. In Jordan alone, there are approximately 1.26 million Syrian residents; 656,900 of them are registered as refugees with the UNHCR, and approximately 600 thousand who are not registered [1,2,3]. These refugees are receiving healthcare from governmental and some Non-Governmental Organizations (NGOs). Cardiovascular disease (CVD) is very prevalent among Syrians [4]. However, there is no literature on CVD in adult Syrian refugees. It is known after World War II that situations such as in Syria create major stress and lead to an increased cortisol baseline level and low cortisol response to stress. If, under such circumstances, a myocardial infarction occurs, the mortality rates rise a fourfold level in comparison to normal population as described in the LIVICordia study. The risk to develop CVD in this population was increased in the long-term [5]. Migration and belonging trauma of Syrian refugees, additional socioeconomic problems generate an enormous amount of stress and thus is causing cardiovascular disease.

In this research we are observing cardiovascular disease in adult Syrian refugees referred to the Jordan University Hospital. This study includes the presenting CVD in the population, its treatment choices, prognosis, outcome, and the fund source status. We also followed the patients to assess whether they actually received the management offered, their quality of life after treatment, and their mortality rate.

### Where do you find Syrian Refugees in Jordan?

About 1.26 million Syrian people live on Jordanian soil [2], which constitutes around 13.2% of population living in Jordan [2]. Of these 1.26 million, only 656,990 are registered refugees [1]. Registered Syrian refugees can be found in two types of areas in Jordan: urban areas and camps. The major portion of refugees is found in urban areas, where around 515,923 (78.5%) of refugees reside, while the remaining 140,990 (21.5%) reside in refugee camps [1]. The Amman and Irbid governates have the highest portion of refugees at 27.3% and 20.8%, respectively. Al-Zatari camp (79,660) and Al-Azraq camp (53,856) have the largest population of Syrian refugees among all refugee camps in Jordan and are hosting around 12.1% of all Syrian refugees in Jordan [2].

### How do Syrian refugees receive health care in Jordan?

Healthcare service access for registered refugees is different according to the residence: refugees registered at refugee camps have access to mobile health clinics set up by the Jordanian Ministry of Health, various nongovernmental organizations (NGOs), and UN agencies [6].

Refugees living outside camps primarily access existing governmental health care centers, some of which are funded and maintained by NGOs and UN agencies. For primary health care, centers both inside camps and major cities provide services to Syrian refugees free of charge or through a cost-sharing mechanism, organized by the UNHCR through the Jordan Health Aid Society [6,7].

When secondary or tertiary health care is sought (such as cardiovascular service), patients must be referred to centers located in Jordan’s major cities. This is a tedious and taxing process that requires time and effort. The situation escalated after these patients reached the healthcare provider and were advised to undergo complex medical or surgical management. The costs of such treatments are not always covered by Jordanian host government, NGOs, or the UNHCR [6]. In our experience, we rely on charity funds, either individual or organizations, to cover the management cost in our hospital [6,7].

## Methods

This is a retrospective cross-sectional study including Syrian refugee adults (aged 18 and above), who were referred to the Cardiology and Cardiovascular Surgery Departments at Jordan University Hospital (JUH) between January 2012 and October 2016. The files of these patients were thoroughly reviewed, including laboratory chemistry, cardiovascular imaging, and other diagnostic modalities. A review of the history, physical examination, electro- and echocardiography studies, and cardiac catheterization were performed prior to the procedures as standardized at JUH. The planned and delivered therapies were recorded; the funding sources were also registered. Patients were followed until January 2017.

## Results

Between January 2012 and October 2016, 969 Syrian refugees were referred to cardiology and cardiac surgery clinics at Jordan University Hospital; 686 (72.2%) of them were males and 283 (27.8%) were females. The mean age at presentation was 55.6 years with a median of 56 years (range 19 to 93 years). Table 1 in this study, 134 patients lived in refugee camps (13.8%) and 835 patients lived outside camps (86.2%). Of the patients studied, 584 patients (60.2%) had arterial hypertension, 308 patients (31.7%) had diabetes mellitus, 281 patients (28.9%) presented with dyslipidemia, and 237 patients (24.4%) were smokers. Table 2 shows the findings with male and female distribution.

**Table 1:** Age distribution of registered refugees.

Age	Male%	Female%	All%	All

60+	1.6	2.2	3.8	24,650
18–59	21.7	23.6	45.3	297,539
0–17	26.2	24.8	51	334,724

**Table 2:** Cardiovascular disease; risk factors and their prevalence.

Risk Factor	Number (%)	Male	Female

Hypertension	584 (60.2%)	69.1%	30.9%
Diabetes Miletus	308 (31.7%)	65.6%	34.4%
Dyslipidemia	281 (28.9%)	71.9%	28.1%
Smoking	227 (24.4%)	82.4%	17.6%

Cardiac catheterization was performed for 828 patients (85.4%) from which 675 patients (69.6%) were diagnosed to have CAD. Of them, 120 had single vessel disease, 182 had two vessel disease, and 373 had three vessel diseases. There are 20 patients, who presented with a valvopathy (10 aortic and 10 mitral valves, 2%). One patient presented with atrial myxoma. There were four patients who demonstrated dysrhythmia as a single diagnosis, 14 (1.5%) suffered from peripheral vascular disease, and two patients had aortic disease.

Table 3 shows the vessel distribution of the CAD group.

**Table 3:** The coronary vessels affected.

Artery	Number(%)

Left Main	75 (7.9%)
LAD	597 (62.9%)
Diagonal	165 (17.3%)
Ramus	22 (2.3%)
Cx and OM	504 (53.1%)
RCA	501 (52.7%)

### Follow-up, Therapy and Fund

Of the whole patient’s population, 489 patients needed interventions in form of PCI or surgery.

We were able to contact and follow Table 4 up 50.8% of our patients. Of those patients and relatives, we were able to contact, 29 had died (2.9%). Eighteen patients (1.8%) (twelve males and six females) died during in hospital treatment (shown in Table 5), whereas eleven patients (1.13%), seven males and four females, died after discharge from hospital.

**Table 4:** Recommended interventions.

Procedure	Number of patients offered treatment

AVR	9
MVR	9
CABG	50
AVR + CABG	1
MVR + CABG	1
PCI	408
PCI VS. CABG	5
PACE MAKER	2
MYXOMA + MVR	1
SVT ABLATION	1
Aortic Disease	2
Total	489

**Table 5:** In hospital mortality causes.

Number	Cause

6	MI
3	Sepsis
3	Renal Failure
5	Heart Failure
1	Post operative

Of 969 patients, 416 were diagnosed to be in need of interventional cardiovascular procedures, and only 299 (71.7%) of them were done. In an additional 50 patients, coronary surgery was indicated. Out of them, surgery was performed in only 14 cases (28%). Twenty patients suffered from valvular heart disease (eight single valves and two cases of valves combined with CABG); valve surgeries were indicated and only eight were performed, as explained in Table 6.

**Table 6:** Offered and provided treatment.

Procedure	Treatment offered	Treatment provided

AVR	9	2
MVR	9	5
CABG	50	13
AVR + CABG	1	0
MVR + CABG	1	1
PCI	408	296
PCI VS. CABG	5	0
PACE MAKER	2	2
MYXOMA + MVR	1	1
SVT ABLATION	1	1
Aortic Disease	2	1
Total	489	322 (65.8%)
Surgical	73	23 (31.5%)
Interventional	416	299 (71.8%)

Funding was mostly supplied by the Jordanian Health Aid Organization; 269 patients received funding from the Jordanian Health Aid Organization, 21 patients depended on their own (through charity providers), 11 patients received funding from NGOs, 14 patients from charities (through personal effort of the treating physician), while seven patients received healthcare outside Jordan in several countries: Canada, Germany, Qatar, and UAE. Of the patients studied, 63 were denied any form of funding.

## Discussion

Since the beginning of the Syrian conflict in 2011, 4.8 million Syrians have been displaced from their country [1]. Of these refugees, 1.26 million reside in Jordan, 656,900 of them are registered with UNHRC, 79,660 live in Al Zaatari camp in the Mafraq Governate, 53,856 in Azraq camp, 7,474 in Murijep al Fhoud camp in the Zarqa governate, 670 in King Abdullah Park, and 311 in Cyber City in the Irbid Governate [1,2]. Registered and nonregistered refugees living outside camps are a vastly larger population than those living in refugee camps, and often face more difficulty receiving healthcare [8]. They rely on the health infrastructure already present in their host communities, and in our investigation, are contributing to an increased pressure on the Jordanian government to provide healthcare for refugees and Jordanians alike [9].

Unlike previous refugee crises, Syrian refugees are dying from non-communicable diseases (NCDs) more than infections [10]. NCDs are killing as many Syrians as bombs and bullets are [10]. Since the rates of hypertension, diabetes mellitus, and CVD have been reported to have the highest prevalence of non-communicable diseases in Syrians, it is imperative to determine whether Syrian refugees in Jordan are given adequate healthcare [4].

Heart disease has been a significant cause of morbidity and mortality in the Syrian adult population. It has been reported that almost half (45%) of deaths in Syria were caused by cardiovascular disease (CVD) before the beginning of the Syrian conflict and mass emigration [4]. About 4.1% (7.5% of those 40–59 years old and 21.4% of those older than 60 years of age) of adult Syrian refugees have some form of CVD [11].

Furthermore, one has to consider the long-term consequences for the refugee generation itself as well as the epigenetic effect: first, consider undertaking primary prevention to protect Syrian refuges from cardiovascular disease, such as giving them Aspirin to protect them from CVD, and second, consider that they will pass down the health-related issues (cardiovascular risk factors and stress reaction and consecutively the predisposition for CVD) to their offspring and thus generate the necessity of treatment in generations to come.

In this study, we followed 969 Syrian refugees who presented to the JUH with CVD over a 45-month period.

JUH is a tertiary care center, which receives the most referrals of Syrian refugees. More than two-thirds of the referred 949 patients (69.6%) were diagnosed with coronary artery disease (CAD). This is a higher percentage than the published figures of CAD in the general Syrian population [4] or in similar countries such as Jordan [12]. This may be explained by the fact that JUH is a tertiary hospital and this number represents referred patients, and not a screen of the normal population. In addition, the lack of a proper primary healthcare system and provided service has put this patient population in situations that have probably accelerated their primary disease.

Of the patients, 489 needed further interventions, whereas 480 were treated medically and sent back home with no proper communication with their primary healthcare provider due to lack of organized referral and follow-up system mainly based on financial causes, and to lack of a unified body that regulates the healthcare system for these refugees.

Of the 489 patients who were offered further management, only 322 (65.8%) actually received it due to lack of funding. A further breakdown of the numbers showed that 73 patients were offered surgical management and 416 were offered nonsurgical intervention, out of which only 23 (31.5%) and 299 (71.8%) respectively actually received it. This vast difference in support between the surgical and nonsurgical groups is not based on the medical indication for either surgical or interventional procedure, but rather due to funding and the existing financial disparities between surgical and interventional treatment options in the Jordanian healthcare system. In addition, funding groups are always afraid of the costs generated by complications. They are more frequent in surgery. That leads to a certain tendency of the foundations to give their support rather towards nonsurgical intervention.

Of the patients, 408 needed PCI and 50 needed coronary artery bypass surgeries (CABG). Of the 408 coronary percutaneous stent implantations that were indicated and recommended, only 296 were done (72.5%), while only 13 of the 50 (26%) recommended CABG were done. There were 21 patients who had valvular heart disease who needed surgery, yet only nine of the recommended 21 heart valve surgeries (42.9%) were performed. This can be explained by the long and tedious process of gaining funding approval. This process can take months to a year and is often delayed, and sometimes denies necessary procedures, as in these demonstrated cases [6].

As a result, 37 CABG surgeries, 112 percutaneous coronary interventions, and 12 valvular surgeries were not done; even worse, 65 patients from our cohort were denied for any type of funding. These numbers indicate that patients are being put at an unacceptable risk of morbidity and mortality. More initiative has to be undertaken and needs to be done to provide faster funding process and consecutively, funding and treatment for such cases. In addition, these very sick refugees seek asylum or immigration to other countries, such as Europe, hoping to find affordable medical care, which will make their journey ever riskier and more dangerous [13].

A greater primary healthcare effort is necessary and demanded to control CVD risk factors to potentially lower these numbers and reduce the strain on Jordanian Health Services.

In our study, half of the patients were previously diagnosed with hypertension, one third with diabetes mellitus, one third with dyslipidemia, and a quarter who were smokers.

Other study showed 15% of Syrian refugees who have NCDs didn’t and don’t receive any care, indicating that controlling these CVD risk factors in a primary healthcare setting may reduce complications such as CAD [14].

Follow-up was not possible for 49.2% of patients in this study. This is most likely due to multiple factors, including patients having no money to pay for transportation, no means to pay for healthcare, and loss of contact with the patient due to changes or loss of contact addresses [6]. This proportion of lost follow-up is high compared to the 12% of follow-up lost in Al-Ammouri et al.’s study, most likely because they studied CVD in children. Raising funds for children’s aid probably attracts more response than in adults; another cause could be a higher number of adults seeking asylum or immigration to other countries than in children.

Such a high rate of loss of follow-up (49.2%) is dangerous and sometimes fatal for patients. Patients with CVD are usually discharged with an anticoagulation and/or antiplatelet regimen as well as medication to treat CVD risk factors if prevalent. Follow-ups are needed to ensure the patient’s compliance and access of medication. Some drugs, such as Warfarin, need regular monitoring. In addition, regular follow-ups are essential for any adjustments after surgical or nonsurgical interventions to prevent complications and to gage the recovery of patients from treatment. Low follow-up rates could translate into higher mortality and morbidity for CVD patients.

In our study, most funding was provided by the Jordanian Health Aid Organization, and still about 158 (33% of intended to treat) patients who needed funds were denied and could not receive the adequate and indicated medical treatment. More funds are needed to provide healthcare for this already vulnerable population. The Jordanian Ministry of Health provides access to healthcare for Syrian refugees outside camps, but resources are dwindling [15]. Even with the help of NGOs and a few physicians from the private sector, the healthcare Syrian refugees receive is not sufficient. Jordanian healthcare is being negatively affected, with only 23 doctors per 10,000 of the population being available in 2013 [16]. General tensions regarding Jordanian funds are on the rise, healthcare being the main reason [15]. The government of Jordan is widely supportive to assist Syrian refugees; however, there is a need for more funds to help Jordan upgrade its facilities to meet the many health needs of Syrian refugees [16]. More support from the international community is needed to deal with the pressure exerted on Jordan’s healthcare system by Syrian refugee health needs [10,16,17].

Some limitations in this study include the single center experience described exclusively by the Jordan University Hospital, which affects the generalizability of these results to the rest of the Syrian refugees in Jordan and other host countries. In addition, losing contact with half of the patients after treatment prevents more accurate estimates for their ensuing quality of life and mortality rates. However, due to the lack of data on CVD in Syrian refugees, this study still provides insight into the current situation of CVD and its risk factors as well as the need for increased healthcare funding.

## Conclusion

This study demonstrates that cardiovascular disease is common among Syrian refugees in Jordan and is demonstrating a higher percentage in comparison to the normal Syrian population. Increased efforts in primary healthcare are needed to adequately treat CVD risk factors and thus prevent future disease progression and its related complications. The number of Syrian refugees unable to pay for life-saving procedures in this study is unacceptably high. Jordan is in need of increased financial support from the international community to fund all levels of its healthcare system to provide Syrian refugees with appropriate healthcare support. Further efforts must be made to maintain regular follow-ups with CVD patients to ensure better quality of life and health outcomes.
